# Neuropathic-like symptoms and the association with joint-specific function and quality of life in patients with hip and knee osteoarthritis

**DOI:** 10.1371/journal.pone.0199165

**Published:** 2018-06-14

**Authors:** Tim Blikman, Wietske Rienstra, Jos. J. A. M. van Raay, Baukje Dijkstra, Sjoerd K. Bulstra, Martin Stevens, Inge van den Akker-Scheek

**Affiliations:** 1 University of Groningen, University Medical Center Groningen, Department of Orthopedics, Groningen, The Netherlands; 2 Martini Hospital Groningen, Department of Orthopedics, Groningen, The Netherlands; 3 Medical Center Leeuwarden, Department of Orthopedics, Groningen, The Netherlands; Tokai Daigaku, JAPAN

## Abstract

**Objective:**

There is an association between osteoarthritis-related pain severity and function, yet clear evidence about the sole influence of neuropathic-like symptoms on joint function and health-related quality of life (HRQoL) is lacking. Previous studies among knee OA patients show an association between neuropathic-like symptoms, lower functional status and lower quality of life, however analyses were unadjusted or had limited adjustment for influential covariates like pain intensity. The aim of this study was therefore to determine the influence of neuropathic-like symptoms—adjusted for multiple influential covariates—on joint-specific function and HRQoL in hip and knee OA patients.

**Methods:**

In this observational study 255 patients (117 with hip OA and 138 with knee OA) completed the modified painDETECT questionnaire (mPDQ) to identify subjects with neuropathic-like symptoms (mPDQ score>12, possible neuropathic pain [NP] phenotype). The WOMAC and the RAND-36 were used to asses respectively function and HRQoL. Results were adjusted stepwise for age, sex and BMI (Model 1); back disorder, painful body regions, comorbidities and previous surgery (Model 2); and pain intensity and analgesic usage (Model 3).

**Results:**

A possible NP phenotype was experienced by 37% of hip and 46% of knee OA patients. Final model 3 analysis revealed that hip OA patients with neuropathic-like symptoms scored significantly lower on pain-related aspects of HRQoL (ΔRAND-36 bodily pain: 6.8 points, p = 0.047) compared to patients with the unlikely NP phenotype. In knee OA patients, a possible NP phenotype was associated with diminished joint function (ΔWOMAC domains ranging 7.1 to 10.5 points, p<0.05) and more deficits on the physical functional aspect of HRQoL (ΔRAND-36 physical functioning: 6.8 points, p = 0.016).

**Conclusion:**

Neuropathic-like symptoms deteriorate the subjective rating of pain-related quality of life in hip OA patients and significantly influence function in knee OA patients.

## Introduction

Hip and knee osteoarthritis (OA) is among the leading causes of disability around the world. This degenerative joint disease has a major impact on quality of life, especially in terms of pain and functional disability [1]. Around 1.3 million people in the Netherlands suffer from OA [2]. Worldwide estimates for symptomatic OA are around 10% of men and 20% of women aged over 60 [3].

As indicated, a common and invalidating key symptom of OA is pain [4]. Multiple studies show that the OA pain experience is not solely nociceptive: about 20% of hip and 20–67% of knee OA patients present neuropathic-like symptoms [5–12]. In OA, such symptoms probably arise from structural changes in joint innervation and neural changes at several levels of the nervous system [13,14]. Clinical features may include hyperalgesia, paraesthesia, burning pain, allodynia and numbness [4]. These neuropathic-like symptoms are not sufficiently treated by conventional first-line nociceptive analgesics, as their effect sizes are limited in OA [15,16].

Despite the clear association between OA-related pain severity and functional limitations [17,18], unambiguous evidence about the association between neuropathic-like symptoms, joint-specific function and health-related quality of life (HRQoL) is lacking. These associations could stress the need for customized conservative OA treatment for OA patients with neuropathic-like symptoms [13]. This would benefit primary caregivers as well as orthopedic surgeons, as optimal conservative treatment could enhance function and HRQoL, eventually delaying the process to total joint replacement.

Although in the available literature more neuropathic-like symptoms are associated with a lower functional status [6,9,11,12,19,20] and diminished (HR)QoL among knee OA patients [11,19], study results are mostly unadjusted or only limitedly adjusted for important covariates. Adjustment is highly necessary, as previous studies show us that several covariates are strongly related with neuropathic-like symptoms [5,6,9,11,19], diminished joint function and quality of life [19,21]. Taking into account factors such as history of previous joint surgery, basic pain intensity and pain at multiple body regions is thus essential if one is interested in the sole influence of neuropathic-like symptoms on joint-specific function and HRQoL.

The aim of the study is therefore to determine the influence of neuropathic-like symptoms, adjusted for multiple important influential covariates, on joint-specific function and HRQoL in a hip and knee OA patient cohort.

## Materials and methods

### Study participants and procedure

**Fig 1:**
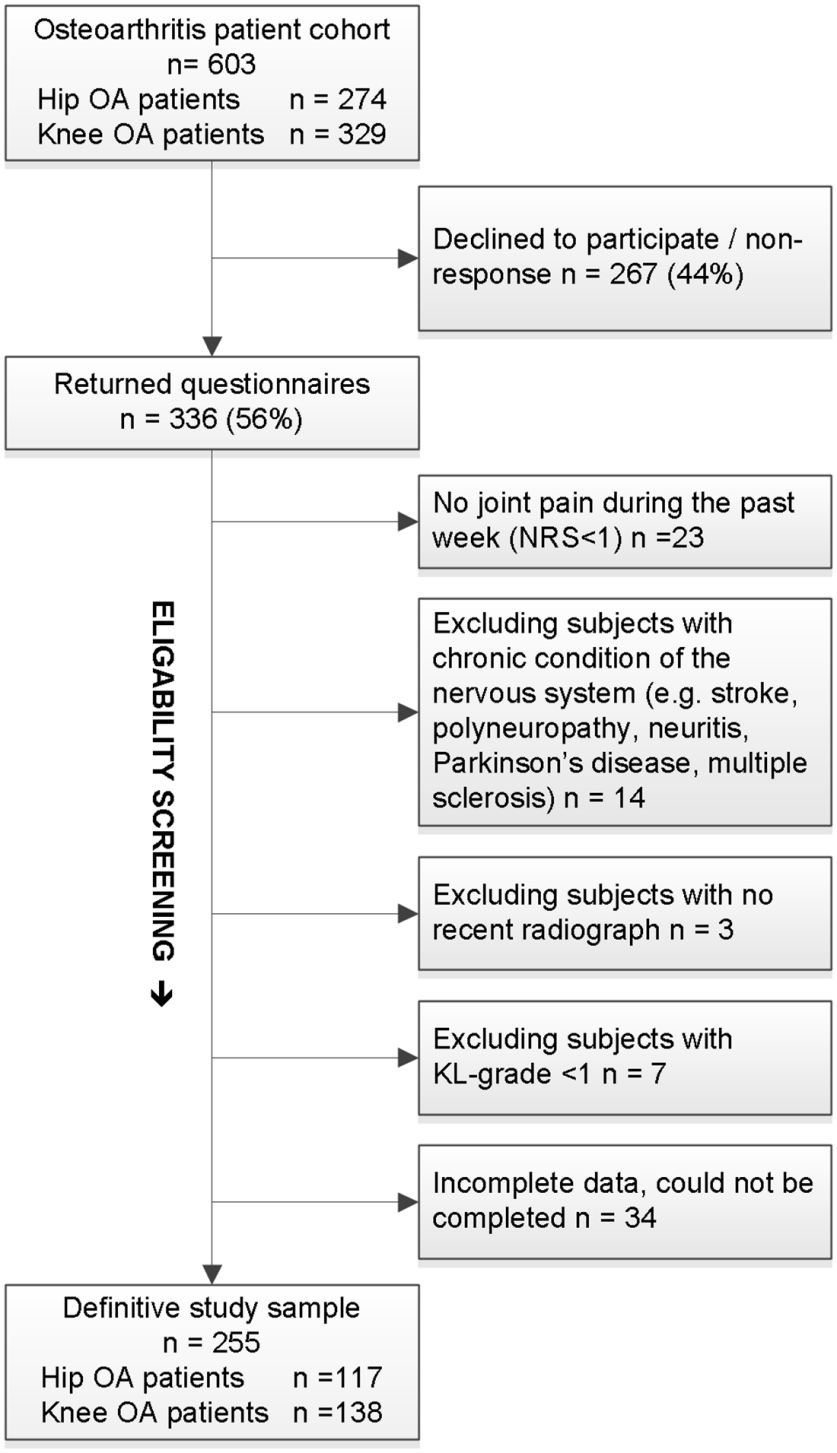
Study recruitment diagram (flowchart).

For this cross-sectional observational study a cohort of 603 patients was obtained from hospital registration lists and invited by mail to fill out a questionnaire. All were adult patients (age >18 years) that were diagnosed with primary hip or knee OA by their treating orthopedic surgeon and visited the orthopedic outpatient clinics of University Medical Center Groningen, Martini Hospital Groningen or Medical Center Leeuwarden, all in the Netherlands, between July 2013 and May 2014. The responders were subsequently checked for additional exclusion criteria. Exclusion criteria were inflammatory arthritis (e.g. rheumatoid arthritis), hip/knee surgery within the last six months, chronic conditions of the nervous system, cognitive or psychiatric disorders, no joint pain during the past week, and inadequate understanding of the Dutch language. Patients were also excluded if they showed no sign of radiographic degeneration as defined by the Kellgren-Lawrence [KL] classification grade <1 on the anteroposterior radiograph. The typical radiographic OA severity cutoff point (KL≥2) was not used, due to the known discordance between radiographic severity and pain [22]. Furthermore, excluding subjects with minor degeneration on the radiograph could bias the results as neural changes (like central sensitization) are especially common among patients who report high levels of clinical pain in the absence of moderate-to-severe radiographic OA [23]. Data was collected with the approval of the local medical ethics committee of University Medical Center Groningen (no. METc2013/515). Informed consent was considered obtained if the patient granted our request to participate by returning the completed set of questionnaires. Patients were informed of this way of obtaining consent by the invitation letter. See Fig 1 for a flowchart.

### Measures

#### Radiographic assessment

Radiographic severity was determined from the most recent anteroposterior radiograph. These radiographs were all taken as part of the patient’s usual care within one year prior to the questionnaire survey. Radiographs were rated by a single observer (T.B.) using the KL grade classification (I-IV) [24]. Rating was done in one session and the rater was blinded to the clinical status of the patient.

#### Patient characteristics

Gender, age, height and weight, family status (living alone/not living alone), highest level of education, duration of joint pain (in months), awaiting joint replacement (index-joint), previous surgery index-joint area, number of significant painful joint/body regions (yes/no answers for 13 regions: head, neck, shoulder, arm, hand, thorax, belly, upper spine, lower spine, hip [non-index], knee [non-index], ankle, foot), comorbidities (yes/no answers for nine groups of diseases associated with diminished quality of life and mortality [25]: migraine, hypertension, pulmonary disease, chronic bowel disorder, severe or persistent back disorder, diabetes, myocardial infarction, severe cardiac condition, cancer) and analgesic consumption.

#### Neuropathic-like symptoms

Neuropathic-like symptoms were determined by means of the self-reported modified painDETECT questionnaire (mPDQ) [26]. It is composed of seven items evaluating pain quality, one item evaluating pain pattern, and one item evaluating pain radiation. The total score is an aggregated score ranging from -1 to 38. The 12-point cutoff point was used to discriminate unlikely NP phenotype patients (mPDQ≤12) from possible NP phenotype patients (mPDQ>12). The PDQ has been validated in a heterogeneous group of low back pain patients, with 80% sensitivity and specificity (cutoff point PDQ≥18, reference: two pain physicians’ diagnoses) [27]. Only one small validation study among knee OA patients was done, finding a sensitivity of 50% and a specificity of 74% for the cutoff point of >12 (reference: quantitative sensory testing exam) [8]. The Dutch mPDQ hip/knee proved to be reliable [26] and has adequate structural and construct validity [28].

#### Joint pain intensity

Average pain intensity in the past week within the index joint was obtained by an 11-point NRS, with 0 representing “no pain” and 10 representing “pain as bad as you can imagine”. The NRS showed to be highly reliable in rheumatic patients (r = 0.95–0.96) [29]. For construct validity, the NRS correlated strongly with the visual analogue scale in patients with rheumatic and other chronic pain conditions (r = 0.83–0.96) [29,30].

#### Joint-specific patient-centered functional outcomes

Participants completed the Hip disability and Osteoarthritis Outcome Score (HOOS) [31] and knee OA patients the Knee injury and Osteoarthritis Outcome Score (KOOS) [32]. Standardized response options are given and each question is scored on a 5-point Likert scale. Subsequently, to make the hip and knee scores comparable and uniform, Western Ontario and McMaster Universities Osteoarthritis Index (WOMAC) scores were calculated from the HOOS or KOOS according to the HOOS/KOOS manuals [33,34]. The WOMAC consists of 24 items divided into three dimensions: pain (5 items), stiffness (2 items) and physical function (17 items). Standardized response options are given and each question is scored on a 5-point Likert scale. For each dimension a normalized score (0–100 range, worst to best) was calculated. The Dutch version of the WOMAC, as well as the HOOS and KOOS, were proven to be reliable and valid [35–37].

#### Health-related quality of life

The RAND 36-item Health Survey (RAND-36) is a generic health status questionnaire. It consists of 36 questions organized into eight multi-item scales: physical functioning (PF), role-physical (RP), bodily pain (BP), general health (GH), vitality (VT), social functioning (SF), role-emotional (RE) and mental health (MH). Each raw scale score is transformed into a linear 0–100 scale (worst to best). The higher the score, the less disability. The Dutch-language version was proven to be practical, reliable and valid [38].

### Statistical methods

Statistical analyses were conducted by using IBM SPSS (V.23). Analyses were conducted separately for hip and knee OA patients. Descriptive statistics were used to describe the study sample. Differences between the unlikely NP phenotype and the possible NP phenotype were analyzed in case of continuous variables with a Student t-test. A Mann-Whitney U-test was performed in cases of skewness (normality checked by histogram). Fisher’s exact test was used for non-continuous data. To estimate the influence of neuropathic-like symptoms on joint-specific function (WOMAC) and health-related quality of life (RAND-36) an ANCOVA analysis was performed, enabling adjustment for the potential influences of the multiple covariates. After a check for basic assumptions like collinearity (variance inflation factor) and homoscedasticity three separate stepwise models were created. Model 1 added the three basic variables of age, sex and BMI to the crude data. Model 2 added severe or persistent back disorder (Yes/No), painful joint/body regions (/13), comorbidities (/9) and previous surgery in index-joint (Yes/No) to Model 1. Model 3, the fully adjusted model, added pain intensity and analgesic usage (Yes/No) to Model 2, to gain insight into the influence of basic pain intensity and analgesic usage on neuropathic like-symptoms. Dependent variables in the ANCOVA analyses were separate WOMAC and RAND-36 domains. A p-value <0.05 was considered statistically significant.

## Results

### Participants

The definitive study sample consisted of 255 subjects, 117 primary hip OA and 138 primary knee OA patients. Patient characteristics are displayed in Table 1. No statistically significant differences were observed between the definitive study sample (n = 255) and the non-participants (n = 348) on age (p = 0.087) and gender (p = 0.869). A possible NP phenotype was experienced by 37% of hip and 46% of knee OA patients. Both hip and knee OA patients with a possible NP phenotype had a higher BMI (p = 0.033 / p = 0.006) and more pain-related characteristics like higher pain intensity (p<0.001 / p<0.001) and more painful joint/body regions (p<0.001 / p = 0.05) than the unlikely NP phenotype group. Solely in the hip OA group, statistically significantly more patients with a possible NP phenotype were experiencing back disorders (p = 0.045) and awaiting joint replacement (p = 0.017) than hip OA patients with an unlikely NP phenotype. Exclusively in knee OA patients the duration of joint pain was higher among patients with a possible NP phenotype than among those with an unlikely phenotype (p = 0.001). Both in hip and knee OA patients no differences were observed between the two pain phenotypes on radiographic severity (p = 0.343 / p = 0.876) and history of previous surgery in the index-joint (p = 0.999 / p = 0.131). Additionally, none of the patients reported usage of non-conventional analgesics (e.g. centrally-acting analgesics like antidepressants to address neuropathic pain-like symptoms).

**Table 1:** Characteristics of study participants^†^.

Characteristics	Hip OA patients (n = 117)			Knee OA patients (n = 138)		
	Unlikely NP (n = 74)	Possible NP (n = 43)	P-value	Unlikely NP (n = 75)	Possible NP (n = 63)	P-value
Age, years	66.6 ± 7.5	67.7 ± 8.4	0.471	63.0 ± 10.4	60.6 ± 10.6	0.170
Female, No. (%)	42 (56.8)	32 (74.4)	0.074	37 (49.3)	35 (55.6)	0.497
BMI (kg/m^2^)	26.6 ± 3.74^a^	28.5 ± 4.9^b^	0.033*	27.5 ± 4.8	30.1 ±6.0^a^	0.006*
Single person household, No. (%)	15 (20.3)	11 (25.6)	0.500	16 (21.3)	13 (20.6)	0.999
High education, No. (%)	21 (28.4)	1 3 (30.2)	0.836	32 (42.7)	14 (22.2)	0.012*
Comorbidities (/9), median (Q1-Q3)^a^	1.00 (0;1)	1.00 (1;2)	0.018*	1.00 (0;2)	1.00 (0;2)	0.170
Back disorder, No. (%)	13 (17.8)	15 (34.9)	0.045*	15 (20.0)	19 (30.2)	0.234
Diabetes, No. (%)	8 (11)	3 (7)	0.744	12 (16.0)	12 (19)	0.659
Cancer, No. (%)	2 (2.7)^a^	4 (9.3)	0.192	3 (4.0)	3(4.8)	0.999
Chronic bowel disorder, No. (%)	3 (4.1)	2 (4.7)	0.999	5 (6.7)	4 (6.3)	0.999
Migraine, No. (%)	6 (8.1)	9 (20.9)	0.082	3 (4.0)	6 (9.5)	0.300
Cardiopulmonary condition (/4)^#^, median (Q1;Q3)	0 (0;1)	0 (0;1)	0.380	0 (0;1)	1 (0;1)	0.599
mPDQ score, median (Q1;Q3)	8 (5;9.25)	16 (14;20)	N.A.	7.00 (5;9)	17.00 (14;21)	N.A.
Mean pain NRS-week (/10)	4.2 ± 2.2	6.0 ± 1.7	<0.001*	3.6 ± 2.4	6.3 ± 1.8	<0.001*
Pain duration, median (Q1;Q3), Mon.	24 (12;58.5)	36 (17;60)	0.346	27 (13;96)	62 (24;144)^a^	0.001*
Awaiting joint replacement, No. (%)	7 (9.5)	12 (27.9)	0.017*	6 (8.0)	8 (12.7)	0.406
Painful joint/body regions (/13), median (Q1-Q3)	2.00 (1;3)	4.00 (2;7)	<0.001*	2.00 (1;3)	3.00 (1;5)	0.015*
Kellgren-Lawrence grade (I-IV)	2.6 ± 0.8	2.4 ± 0.8	0.343	2.3 ± 0.7	2.3 ± 0.7	0.876
Previous surgery index-joint, No. (%)	5 (6.8)	2 (4.8)^a^	0.999	17 (22.7)	22 (34.9)	0.131
Analgesic usage, No. (%)	37 (50)	30 (69.8)	0.052	34 (45.3)	37 (58.7)	0.127
Acetaminophen	27 (36.5)	23 (53.5)	0.084	28 (37.3)	31 (49.2)	0.127
Nonsteroidal anti-inflammatory drugs	19 (25.7)	10 (23.3)	0.827	13 (17.3)	15 (23.8)	0.399
Weak opioids	2 (2.7)	1 (2.3)	N.A.	1 (1.3)	-	N.A.
Strong opioids	1 (1.4)	-	N.A.	1 (1.3)	3 (4.8)	N.A.
Others	-	-	-	-	-	-

^†^ Except where indicated otherwise, values are presented as mean ± SD; Unlikely NP = unlikely neuropathic pain phenotype (mPDQ≤12); Possible NP = possible neuropathic pain phenotype (mPDQ>12); BMI = body mass index; mPDQ = modified painDETECT questionnaire; NRS = Numeric Rating Scale; N.A. = not applicable.

^#^ Cardiopulmonary condition (/4): 1) hypertension, 2) pulmonary disease, 3) myocardial infarction, 4) other severe cardiac condition.

^a^ There was 1 individual with missing data on this variable.

^b^ There were 2 individuals with missing data on this variable.

* A P-value <0.05 was considered to be statistically significant.

### Hip OA patients

#### Joint-specific patient-centered functional outcomes (WOMAC)

**Fig 2:**
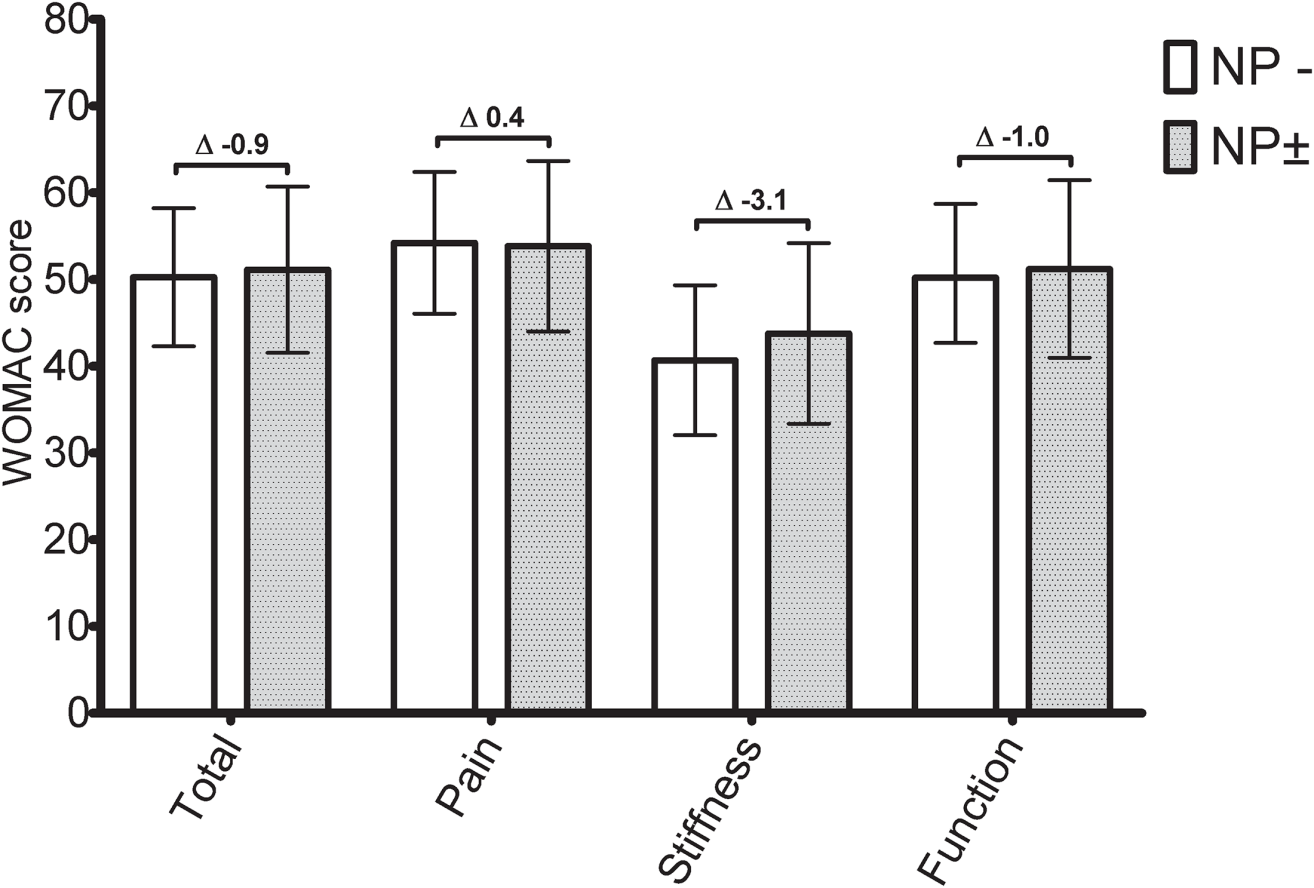
Hip OA Model 3: Fully ANCOVA adjusted WOMAC scores. NP-: unlikely neuropathic pain phenotype (mPDQ≤12). NP±: possible neuropathic pain phenotype (NP±, mPDQ>12). WOMAC score 100 indicates no symptoms/problems and 0 indicates extreme symptoms/problems. Error bar represents the 95% CI (lower-upper limits) of the adjusted mean. Mean adjusted difference is displayed numerically. *: p<0.05.

The crude data showed statistically significant differences between the two pain phenotypes (unlikely versus possible NP, Table 2) in the pain and function domains (except for the stiffness domain). Differences in the crude data were around 10 points for all domains (Table 2). Model 1, which adjusted for age, sex and BMI, did not change the point estimates, even though the confidence interval (CI) in the domain function changed, causing the statistically significant difference to disappear. In model 2, most point estimates dropped and differences were no longer statistically significant. Final model 3 adjustments for pain intensity and analgesic usage (fully adjusted model) caused contrasts between the point estimates of the two phenotypes to disappear due to the possible NP phenotype patients; their WOMAC point estimates increased toward the levels of the unlikely NP phenotype group (Fig 2, Table 2).

**Table 2:** Hip OA crude and stepwise ANCOVA adjusted WOMAC and RAND-36 scores^§^.

Hip OA patients	Crude and Model 3 (Fully adjusted model)		Model 1		Model 2	
	Unlikely NP (n = 74)	Possible NP (n = 43)	Unlikely NP	Possible NP	Unlikely NP	Possible NP
**WOMAC score**						
Total	Crude: 59.5 (55.3–63.7)	49.0 (42.1–55.9)*	58.7 (54.0–63.4)^a^	50.6 (43.9–57.2)^b^*	49.9 (40.8–59.0)^b^	41.7 (31.3–52.1)^c^
	**Model 3**: 50.3 (42.3–58.2)^b^	51.1 (41.6–60.7)^c^				
Pain	Crude: 63.5 (59.2–67.8)	52.8 (45.9–59.7)*	62.6 (57.8–67.4)^a^	53.5 (46.7–60.2)^b^*	53.8 (44.6–62.9)^b^	44.9 (34.4–55.4)^c^
	**Model 3**: 54.2 (46.1–62.4)^b^	53.8 (44.0–63.7)^c^				
Stiffness	Crude: 51.9 (46.7–57.1)	43.9 (38.4–49.4)	51.5 (46.4–56.5)^a^	43.8 (36.7–51.0)^b^	40.4 (30.8–49.9)^b^	35.0 (24.1–45.9)^c^
	**Model 3**: 40.7 (32.0–49.3)^b^	43.8 (33.4–54.2)^c^				
Function	Crude: 59.2 (54.8–63.6)	48.53 (41.1–55.9)*	58.4 (53.4–63.4)^a^	50.5 (43.5–57.5)^b^	49.8 (40.2–59.5)^b^	41.6 (30.6–52.5)^c^
	**Model 3**: 50.2 (42.7–58.7)^b^	51.2 (41.0–61.5)^c^				
**RAND-36 score**						
Physical Functioning	Crude: 56.9 (51.6–62.2)	42.4 (35.1–49.7)^a^*	56.0 (50.8–61.2)^a^	45.9 (38.5–53.4)^c^*	54.4 (35.6–55.2)^b^	37.1 (25.9–48.2)^c^
	**Model 3**: 45.8 (36.4–55.1)^b^	43.9 (32.6–55.1)^c^				
Role-Physical	Crude: 56.1 (46.5–65.7)	25.0 (15–35)*	56.0 (46.7–65.3)^a^	31.2 (18.1–44.2)^b^*	38.2 (20.4–55.9)^b^	16.6 (-3.6–36.8)^c^*
	**Model 3**: 39.1 (22.1–56.2)^b^	27.7 (7.2–48.3)c				
Bodily Pain	Crude: 60.7 (57–64.4)	43.3 (37.5–49.1)*	60.2 (56.1–64.4)^a^	44.2 (38.4–50.0)^b^*	48.7 (41.3–56.2)^b^	34.5 (26.1–43.0)^c^*
	**Model 3**: 49.2 (42.8–55.6)^b^	42.4 (34.7–50.1)^c^*				
General Health	Crude: 64.4 (60.5–68.3)	61.4 (56.6–66.2)	64.0 (60.2–67.9)^a^	63.0 (57.6–68.5)^b^	65.1 (57.8–72.3)^b^	67.1 (58.8–75.3)^c^
	**Model 3**: 65.7 (58.5–72.8)^b^	60.2 (60.5–77.8)^c^				
Vitality	Crude: 65.0 (61.1–68.9)	56.6 (51.1–62.1)*	64.5 (60.4–68.6)^a^	58.40 (52.6–64.2)^b^	61.3 (53.5–69.2)^b^	57.0 (48.1–66.0)^c^
	**Model 3**: 62.1 (54.4–69.8)^b^	59.4 (50.1–68.7)^c^				
Social Functioning	Crude: 85.0 (80.9–89.1)	70.9 (62.5–79.3)*	84.5 (79.4–89.7)^a^	74.0 (66.7–81.3)^b^*	76.9 (66.9–86.9)^b^	68.1 (56.7–79.5)^c^
	**Model 3**: 77.1 (67.4–86.8)^b^	74.2 (62.6–85.9)^c^				
Role-Emotional	Crude: 91.0 (85.2–96.8)	72.1 (60.1–84.1)*	90.4 (83.1–97.7)^a^	75.8 (65.5–86.1)^b^*	86.8 (72.4–101.1)^b^	74.5 (58.2–90.8)^c^
	**Model 3**: 88.0 (73.8–102.2)^b^	76.6 (59.5–93.7)^c^				
Mental Health	Crude: 80.4 (76.9–83.9)	78.1 (73.9–82.3)	80.7 (77.2–84.2)^a^	79.6 (74.7–84.5)^b^	82.8 (76.0–89.6)^b^	83.3 (75.6–91.1)^c^
	**Model 3**: 83.4 (76.8–90.0)^b^	86.3 (78.3–94.3)^c^				

^§^ All values are presented as mean (95% CI)

Crude: Unadjusted values

Model 1: adjusted for age, sex, BMI

Model 2: Additionally adjusted for back disorders, painful body regions, comorbidities and previous surgery (includes model 1)

Model 3: Fully adjusted model, additionally adjusted for pain intensity and analgesic usage (includes model 2)

Unlikely NP = unlikely neuropathic pain phenotype (mPDQ≤12); Possible NP = possible neuropathic pain phenotype (mPDQ>12); WOMAC = Western Ontario and McMaster Universities Osteoarthritis Index; RAND-36 = RAND 36-item Health Survey; WOMAC/RAND-36 score = 100 indicates no symptoms/problems and 0 indicates extreme symptoms/problems

^a^ There was 1 individual with missing data on this variable

^b^ There were 2 individuals with missing data on this variable

^c^ There were 3 individuals with missing data on this variable

* A P-value <0.05 was considered to be statistically significant

#### Health-related quality of life (RAND-36)

Except for the general health and mental health domains, all other domains on the RAND-36 (6/8) displayed statistically significant differences between the two pain phenotypes (unlikely versus possible NP, see Table 2). Statistically significant differences in the crude data ranged from 8.4 (vitality) to 31.1 points (role-physical). Model 1 adjustments did not change point estimates, albeit the difference in the domain vitality was no longer statistically significant. Adjustments made in model 2 for potentially influential covariates like number of comorbidities and experiencing a severe or persistent back disorder resulted in statistically significant differences solely in the domains role-physical (8.7 points) and bodily pain (14.2 points). Additional final adjustment for pain intensity and analgesic usage (model 3) only retained the bodily pain domain; the statistically significant difference was 6.8 points (p = 0.047).

### Knee OA patients

#### Joint-specific patient-centered functional outcomes (WOMAC)

**Fig 3:**
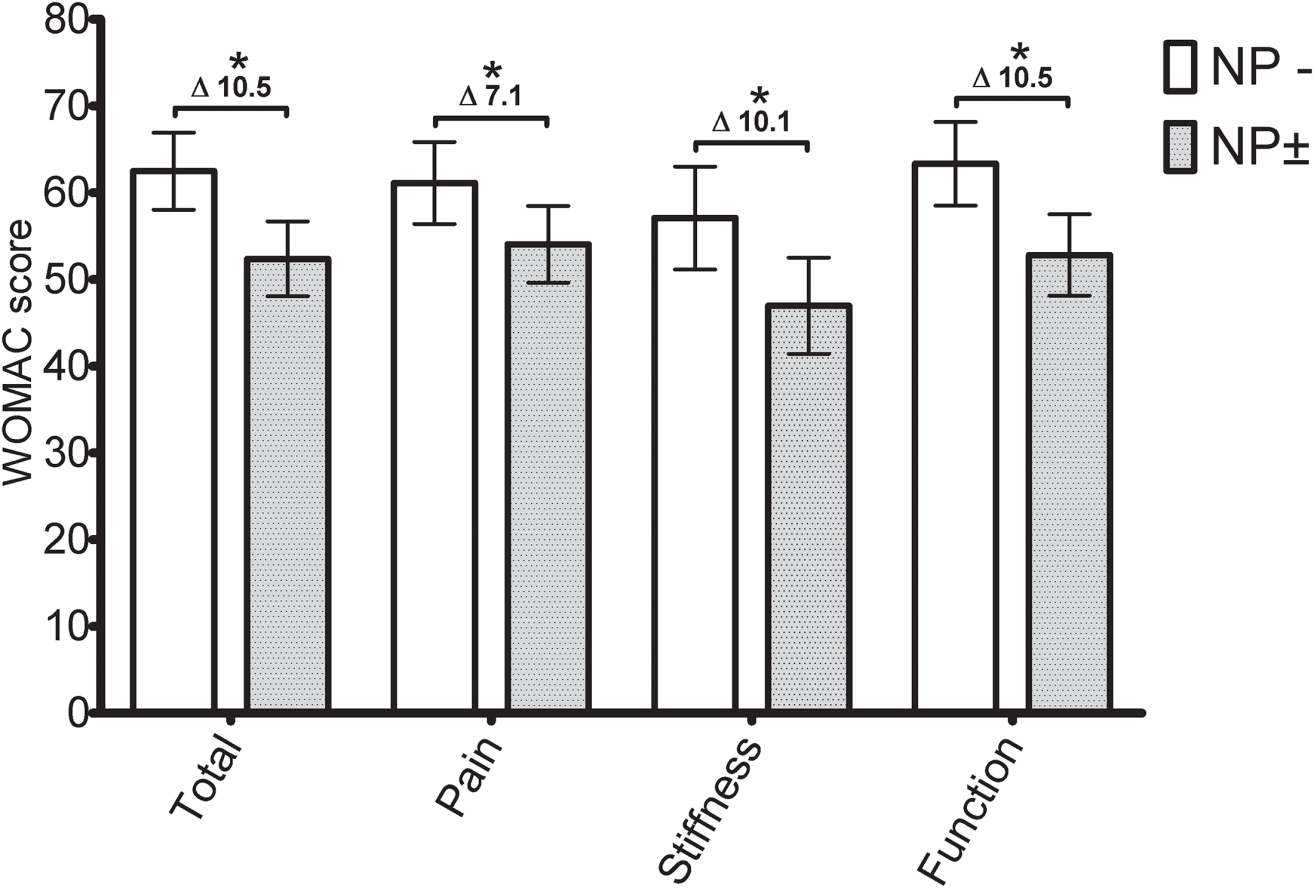
Knee OA Model 3: Fully ANCOVA adjusted WOMAC scores. NP-: unlikely neuropathic pain phenotype (mPDQ≤12). NP±: possible neuropathic pain phenotype (NP±, mPDQ>12). WOMAC score 100 indicates no symptoms/problems and 0 indicates extreme symptoms/problems. Error bar represents the 95% CI (lower-upper limits) of the adjusted mean. Mean adjusted difference is displayed numerically. *: p<0.05.

All domains on the WOMAC displayed statistically significant differences between the two pain phenotypes (unlikely versus possible NP, see Table 3). In the crude data, differences between the point estimates of the two pain phenotypes were roughly twice as large in the knee OA group (knee difference [95% CI] WOMAC total score: 23.8 [17.9–29.6]) compared to the hip OA group (hip OA difference [95% CI] WOMAC total score: 10.5 [2.7–18.2]). Model 1, which adjusted for age, sex and BMI, did not change point estimates. Additional adjustment in model 2 had no influence on point estimates either. Ultimately, final adjustment for pain intensity and analgesic usage (model 3) reduced contrasts between the two phenotypes, from around 20 points (model 2) to around 10 points (model 3). Differences remained statistically significant on all WOMAC domains (Fig 3, Table 3).

**Table 3:** Knee OA crude and stepwise ANCOVA adjusted WOMAC and RAND-36 scores^§^.

Knee OA patients	Crude and Model 3 (Fully adjusted model)		Model 1		Model 2	
	Unlikely NP (n = 75)	Possible NP (n = 63)	Unlikely NP	Possible NP	Unlikely NP	Possible NP
**WOMAC score**						
Total	Crude: 68.4 (64.3–72.5)	44.6 (40.7–48.5)^c^*	67.2 (63.5–71.0)	46.2 (41.9–50.5)^d^*	67.5 (62.6–72.5)	47.8 (43.0–52.6)^d^*
	**Model 3**: 62.5 (58.1–66.9)	52.4 (48.1–56.7)^d^*				
Pain	Crude: 67.7 (63.2–72.2)	45.0 (40.6–49.4)*	66.4 (62.2–70.6)	46.8 (42.2–51.4)^a^*	67.9 (62.3–73.5)	48.0 (42.8–53.3)^a^*
	**Model 3**: 61.1 (56.4–65.9)	54.1 (49.6–58.5)^a^*				
Stiffness	Crude: 62.5 (57.6–67.4)	39.5 (34.9–44.1)*	61.5 (56.8–66.2)	40.6 (35.4–45.8)^a^*	61.8 (55.7–67.9)	42.3 (36.5–48.0)^a^*
	**Model 3**: 57.1 (51.2–63.0)	47.0 (41.4–52.5)^a^*				
Function	Crude: 69.2 (65–73.4)	45.6 (41.5–49.7)^c^*	68.1 (64.2–72.0)*	47.1 (42.7–51.6)^d^*	67.8 (62.6–72.9)*	48.2 (43.1–53.2)^d^*
	**Model 3**: 63.4 (58.5–68.2)	52.8 (48.1–57.5)^d^*				
**RAND-36 score**						
Physical Functioning	Crude: 56.6 (51.6–61.6)^a^	38.1 (33.3–42.9)*	55.5 (50.8–60.1)^a^	39.8 (34.7–44.9)^a^*	55.7 (49.8–61.6)^a^	41.0 (35.5–46.5)^a^*
	**Model 3**: 53.4 (47.3–59.5)^a^	43.7 (38.1–49.4)^a^*				
Role-Physical	Crude: 53.7 (44.3–63.1)	38.3 ± 28.1–48.1^a^*	52.1 (42.5–61.6)	39.3 (28.6–49.9)^b^	56.2 (43.8–68.5)	41.9 (30.2–53.6)^b^
	**Model 3**: 54.5 (41.4–67.5)	44.6 (32.3–57.0)^b^				
Bodily Pain	Crude: 48.8 (44–53.6)	54.1 (49.2–59)^a^	49.7 (44.9–54.4)	53.1 (47.8–58.4)^b^	49.2 (43.0–55.5)	51.7 (45.8–57.6)^b^
	**Model 3**: 50.3 (43.7–56.9)	50.5 (44.3–56.8)^b^				
General Health	Crude: 58.2 (54.6–61.8)	56.7 (52.6–60.8)^a^	57.9 (54.2–61.6)	56.7 (52.6–60.8)^b^	61.0 (56.2–65.7)	58.1 (53.5–62.6)^b^
	**Model 3**: 60.2 (55.2–65.3)	58.0 (53.3–62.8)^b^				
Vitality	Crude: 57 (53.4–60.6)	52.7 (49.1–56.3)^a^	56.4 (52.9–60.0)	53.0 (49.1–56.9)^b^	59.9 (52.3–61.6)	53.2 (48.8–57.6)^b^
	**Model 3**: 57.8 (52.9–62.7)	52.4 (47.7–57.0)^b^				
Social Functioning	Crude: 59.2 (53.9–64.5)	53.0 (47.4–58.6)^a^	58.6 (53.3–63.9)	53.7 (47.8–59.6)^b^	59.8 (53.1–66.5)	54.1 (47.8–60.4)^b^
	**Model 3**: 61.1 (54.0–68.2)	52.9 (46.2–59.6)^b^				
Role-Emotional	Crude: 83.6 (76.3–90.9)	72.6 (62.6–82.6)^a^	82.0 (73.7–90.3)	73.7 (64.4–83.0)^b^	87.4 (76.6–98.2)	77.7 (67.5–88.0)^b^
	**Model 3**: 88.2 (76.8–99.7)	78.5 (67.7–89.3)^b^				
Mental Health	Crude: 68.2 (65.3–71.1)	65.0 (61.5–68.5)^a^	67.6 (64.5–70.7)	65.5 (62.0–68.9)^b^	69.4 (65.5–73.3)	66.9 (63.2–70.6)^b^
	**Model 3**: 69.5 (65.4–73.7)	67.0 (63.1–70.9)^b^				

^§^ All values are presented as mean (95% CI)

Crude: Unadjusted values

Model 1: adjusted for age, sex, BMI

Model 2: Additionally adjusted for back disorders, painful body regions, comorbidities and previous surgery (includes model 1)

Model 3 / Full: Fully adjusted model, additionally adjusted for pain intensity and analgesic usage (includes model 2)

Unlikely NP = unlikely neuropathic pain phenotype (mPDQ≤12); Possible NP = possible neuropathic pain phenotype (mPDQ>12); WOMAC = Western Ontario and McMaster Universities Osteoarthritis Index; RAND-36 = RAND 36-item Health Survey; WOMAC/RAND-36 score = 100 indicates no symptoms/problems and 0 indicates extreme symptoms/problems

^a^ There was 1 individual with missing data on this variable

^b^ There were 2 individuals with missing data on this variable

^c^ There were 3 individuals with missing data on this variable

^d^ There were 4 individuals with missing data on this variable

* A P-value <0.05 was considered to be statistically significant

#### Health-related quality of life (RAND-36)

The crude data displayed two domains in which the two pain phenotypes (unlikely versus possible NP) significantly differed, namely the physical functioning domain (18.5 points) and the role-physical domain (15.4 points). Basic adjustment for age, sex and BMI (model 1) only retained the statistically significant difference in the physical functioning domain (15.7 points). This difference was retained after adjustments made in models 2 (14.6 points) and 3 (9.6 points).

## Discussion

The aim of this study was to determine the sole influence of neuropathic-like symptoms on joint-specific function and HRQoL, in both hip and knee OA patients, while adjusting for multiple known influential covariates. We found that the presence of neuropathic-like symptoms does not interfere with a hip OA patients’ joint function, but does deteriorate the subjective rating of pain-related quality of life. By contrast, knee OA patients who experience neuropathic-like symptoms encounter clinically relevant diminished joint-related function [39] and more deficits on the physical functional aspect of health-related quality of life. As a substantial proportion of patients experienced neuropathic-like symptoms and none reported using analgesics to treat neuropathic-like symptoms specifically, OA patients could benefit from pain phenotype screening and a more customized treatment for their underlying pain mechanism (e.g. centrally-acting analgesics like antidepressants). Hence results of this study have implications for a broad field of OA caregivers, ranging from family physicians to orthopedic surgeons.

The major additional value of this study compared to previous ones in this field is that extensive adjustments for relevant covariates have been made. The effect of adjusting was especially visible in the hip OA group. Without adjusting, deficits on joint function were apparent. However, as these effects were no longer apparent after adjusting, the found deficits in joint-related function were explained by other covariates.

As indicated, our study is the first to extensively adjust for multiple relevant known covariates, therefore results can only be compared with previous studies to a limited extent. Furthermore, studies on this specific topic are lacking in hip OA patients, so no comparison with previous studies was possible at all for hip OA results. When comparing our findings to previous knee OA studies that did not adjust for relevant covariates (or only did minimally), we found comparable large differences in our unadjusted analyses on the WOMAC between the possible and the unlikely NP phenotype group [12,19,20]. A study by Valdes et al. [20]–the only one that adjusted for basic variables (age, sex, BMI)–found nearly identical statistically significant WOMAC differences when compared to our Model 1 results between the possible NP phenotype and the unlikely NP phenotype on all domains. With respect to HRQoL, only the study by Aṣkin et al. [11] used the SF-36 questionnaire and also found a statistically significant difference on the role-physical domain when comparing patients with neuropathic-like symptoms to patients without these symptoms. However, this statistically significant difference was only found in our crude data and not in our adjusted data. This stresses the importance of adjusting for relevant known covariates to gain insight into the sole influence of neuropathic-like symptoms on function and HRQoL.

In this study neuropathic-like symptoms were quite prevalent among hip and knee OA patients; respectively 37% and 46% experienced at least a possible NP phenotype (m PDQ>12), which is in line with previous research [5–11]. The reported prevalences of neuropathic-like symptoms in OA do vary though, likely due to differences in assessment tools and study methodology. In line with literature [6,9,11,19,20], neuropathic-like symptoms were not associated with age or gender in our study, although Valdes et al. [19] reported that knee OA patients with a possible NP-like phenotype were statistically significantly younger than patients with the unlikely NP phenotype. Moreover, radiographic severity did not differ between the two pain phenotypes, which is in line with previous research [6,19,20]. This finding could imply that cartilage degeneration as such is not associated with neuropathic-like symptoms but with a peripheral or centrally augmented pain state (central sensitization [CS]). It is believed that long-term continuous and intense joint-related nociceptive input drives the sensitization process and leads to local and widespread allodynia, and ultimately to generalized central sensitization [40]. This theory is reinforced by our present findings of increased signs of central sensitization in patients with neuropathic-like symptoms. In our study statistically significantly more patients with the possible NP phenotype experienced a high pain intensity in combination with more pain in other joint/body regions (widespread pain), compared to patients with the unlikely NP phenotype.

Strengths of this study are that it focused solely on a general secondary care hip and knee OA population with few exclusion criteria, which benefits external validity. In contrast to most studies, we used the modified version of the painDETECT questionnaire (mPDQ), which is joint- and population-specific and forces OA patients to think about their specific joint within a delimited timeframe, enhancing face and content validity [9]. This is also one of the recommended questionnaires out of the six existing neuropathic pain screening tools [41]. Moreover, the fact that the mPDQ does not require clinical examination facilitates its use by healthcare professionals.

Unfortunately, to date there is no gold standard to detect definitive neuropathic pain in OA. The International Association for the Study of Pain (IASP) requires a demonstrable lesion or disease that satisfies established neurological diagnostic criteria (e.g. imaging, biopsies) [42]. In daily clinical practice it is still impossible to demonstrate nerve alterations in OA. This is why throughout the article we used the term neuropathic-like symptoms. The appropriateness of the used cutoff point could also be questioned (possible NP phenotype; mPDQ>12). However, as our goal was to capture a group in which the neuropathic pain component can possibly be present (mPDQ>12) and a group in which the neuropathic component is unlikely to be present (mPDQ≤12) [27], it does seem appropriate. Additional sub-analyses with the unlikely NP phenotype (mPDQ≤12) against the likely NP phenotype (mPDQ≥18) did not change the study results. The reported relatively low sensitivity of 50% for the cutoff-point of >12 could be interpreted as problematic [8], yet it only means that there is a possibility of a substantial number of false-negatives among the unlikely NP phenotype group. Hence it is quite possible that the contrasts found between the two pain phenotypes are in fact larger than presented in this study. This is reinforced by the adequate specificity of 74%, which means that there are few false-positives in the possible NP phenotype group.

In conclusion, experiencing neuropathic-like symptoms seem to be associated mainly with diminished physical functioning in knee OA patients. In hip OA patients this only deteriorates the subjective rating of pain-related quality of life. Overall, neuropathic-like symptoms seem to be related to more signs of central pain sensitization. OA patients could benefit from pain phenotype screening and a more customized treatment for their underlying pain mechanism (e.g. centrally-acting analgesics like antidepressants). Future longitudinal RCTs are needed to determine whether such treatment enhances function and quality of life in OA patients with neuropathic-like symptoms.

## Supporting information

S1 Dataset Data underlying this study. (SAV)
